# Case Report: A Brodie's abscess in the spine in a female patient treated by percutaneous endoscopic debridement and drainage

**DOI:** 10.3389/fsurg.2025.1711799

**Published:** 2026-01-02

**Authors:** Tsung-Lin Yang, Chen-Pang Huang, Hung-Kang Wu, Yu-Pao Hsu, Ching-Hsiao Yu

**Affiliations:** 1 Department of Orthopedic Surgery, Taoyuan General Hospital, Ministry of Health and Welfare, Taoyuan City, Taiwan; 2 Department of Orthopedic Surgery, Taipei Veterans General Hospital Taoyuan Branch, Taoyuan City, Taiwan

**Keywords:** Brodie's abscess, spondylodiscitis, vertebral osteomyelitis, percutaneous endoscopic debridement and drainage (PEDD), minimally invasive spine surgery, antimicrobial stewardship, thoracolumbar spine

## Abstract

Brodie's abscess is a rare subacute osteomyelitis. It is characterized by a localized, well-demarcated abscess within the bone, typically in the metaphysis of long bones in young male patients. Spine involvement is extremely rare, accounting for only 1% of all cases. Due to the rarity of this condition, there is no established standard treatment. We present a case of a 37-year-old female patient with a Brodie's abscess in her L1–L2 vertebrae who was successfully treated with percutaneous endoscopic debridement and drainage (PEDD) and concomitant percutaneous instrumentation, the necessity of which was underscored by objective stability assessments. The patient's back pain significantly improved after the surgery, and she was able to walk without any support. The patient was discharged on postoperative day 14 and was followed up for 1 year, with no recurrence of the infection. We believe that PEDD is a safe and effective treatment option for a Brodie's abscess in the spine.

## Introduction

In 1832, Sir Benjamin Brodie described localized tibia infections found in adolescents and young adults that were treated with surgical drainage and debridement (1). A Brodie's abscess is characterized by subacute infection, localized mostly in the tibia (48.6%) and femur (31.3%) (2), while those located in the spine are very rare (0.6%). The lesion is usually confined, with a sclerotic rim that interferes with antibiotic delivery, so surgical drainage has been established as an effective treatment of choice (2). Since the minimally invasive concept has been adopted in many surgical fields and there has been advancement in spine endoscope techniques, percutaneous endoscopic debridement and drainage (PEDD) has become a popular method to treat spinal infections in recent years. In this study, we reported a large Brodie's abscess located at the thoracolumbar spine junction that was treated successfully via percutaneous endoscopic spinal surgery.

## Case history

The 37-year-old woman without any systemic medical disease had chronic back pain for 2 years without a trauma history. Before presenting to our outpatient department, she had visited local clinics several times without a conclusive diagnosis. She had received painkillers, but her back pain was intermittent. In this instance, she visited us due to aggravated back pain for 3 months. In addition to constant pain, she suffered from mechanical back pain when changing position. She also denied fever or leg pain. She was very thin, weighing only 35 kg (BMI = 13.6). A physical examination revealed no skin change on her back and no local tenderness. Laboratory data showed a slightly increased C-reactive protein (CRP) level of 1.2 mg/dL, which normalized on follow-up.

**Figure 1:**
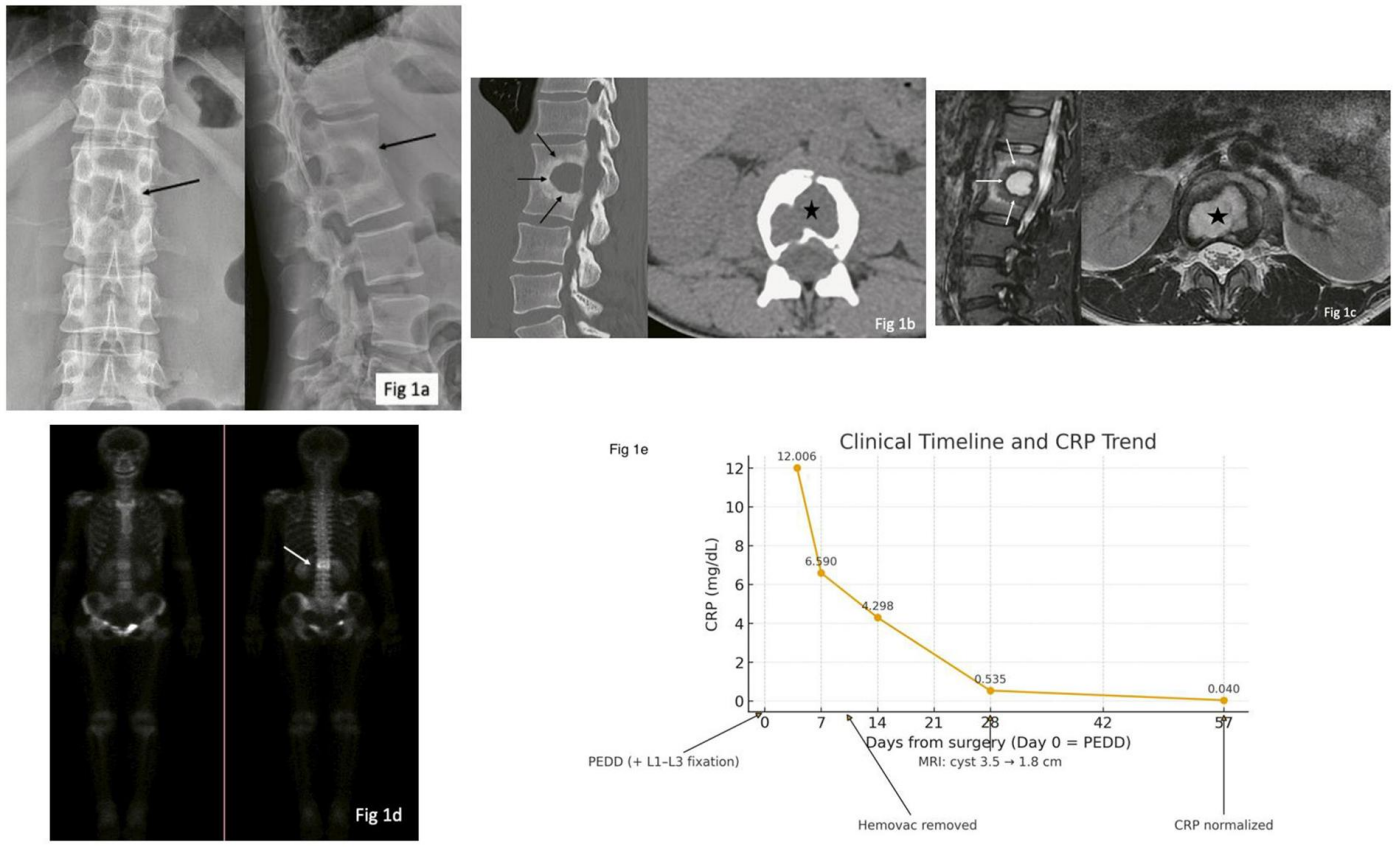
Imaging of the 37-year-old female patient is shown. **(a)** Radiographs of her thoracolumbar spine reveal a cavity (black arrow) in the center-posterior part of the L1/L2 vertebral bodies. **(b)** A CT scan shows fused L1 and L2 facet joints and the cavity (black star), with a peripheral sclerotic rim around the cavity (black arrows). **(c)** MRI reveals fluid accumulation surrounded by a sclerotic zone (white arrows), with increased signal intensity in T2-weighted images without extravasation into the epidural space (black star). **(d)** A whole-body bone scan shows ring enhancement at the L1–L2 level. **(e)** Timeline of the key clinical events and CRP trend.

Radiography of her thoracolumbar spine revealed fused L1 and L2 vertebral bodies, with a huge cavity located within this fused vertebral body (Figure 1a). A CT scan revealed fused L1 and L2 facet joints and vertebral bodies with a sclerotic rim (Figure 1b). MRI showed fluid accumulation surrounded by a sclerotic zone, with decreased signal intensity in both sagittal T1- and T2-weighted images. Besides the inner sclerotic rim, there was an outer edema rim showing hypersignal in T2 and iso-to-low signal in T1. The axial view showed well-confined fluid without extravasation or epidural abscess (Figure 1c). A further whole-body bone scan showed no skip lesions and ring enhancement at the L1 and L2 levels (Figure 1d). An additional panel in Figure 1e summarizes the clinical timeline and CRP trend. Under suspicion of a Brodie’s abscess in the spine, she was admitted for further management. Given that the imaging findings were suggestive of a contained abscess and the initially low CRP, preoperative antibiotics were withheld. Surgical management was chosen as the combined diagnostic and therapeutic approach.

The patient's perspective was a key component of her outcome. She reported profound satisfaction, stating that the endoscopic procedure had finally resolved the severe, chronic back pain that had been debilitating for 2 years. She expressed particular relief with the minimally invasive nature of the surgery, which allowed her to mobilize quickly and return to daily activities with minimal discomfort, an outcome she had not thought possible.

Initial MRI and CT revealed a rim-enhancing intravertebral cavity with a surrounding sclerotic margin at L1–L2, consistent with a contained nidus rather than diffuse phlegmon. The sclerotic rim plausibly limited antibiotic penetration, which explains why surgical source control was necessary and why empiric antibiotic therapy, had it been administered, would likely have failed (3). Differential diagnoses—atypical pyogenic spondylodiscitis, tuberculous/granulomatous infection, cystic tumor, or chronic organized hematoma—were weighed against the imaging and clinical data. Targeted lesion sampling during the index operation yielded methicillin-sensitive *Staphylococcus aureus* (MSSA), which informed downstream antimicrobial choices (4).

The patient's history was reviewed for predisposing conditions. There was no history of diabetes mellitus or hypertension; she had a smoking history of approximately 0.5 pack per day for 7–8 years and denied alcohol use. A portal of entry was not identified. Further investigations to identify a source included blood cultures, which were negative. As the patient was afebrile on admission (BT 37.1°C) and a physical examination revealed no heart murmur, infective endocarditis was deemed unlikely and echocardiography was not performed.

**Figure 2:**
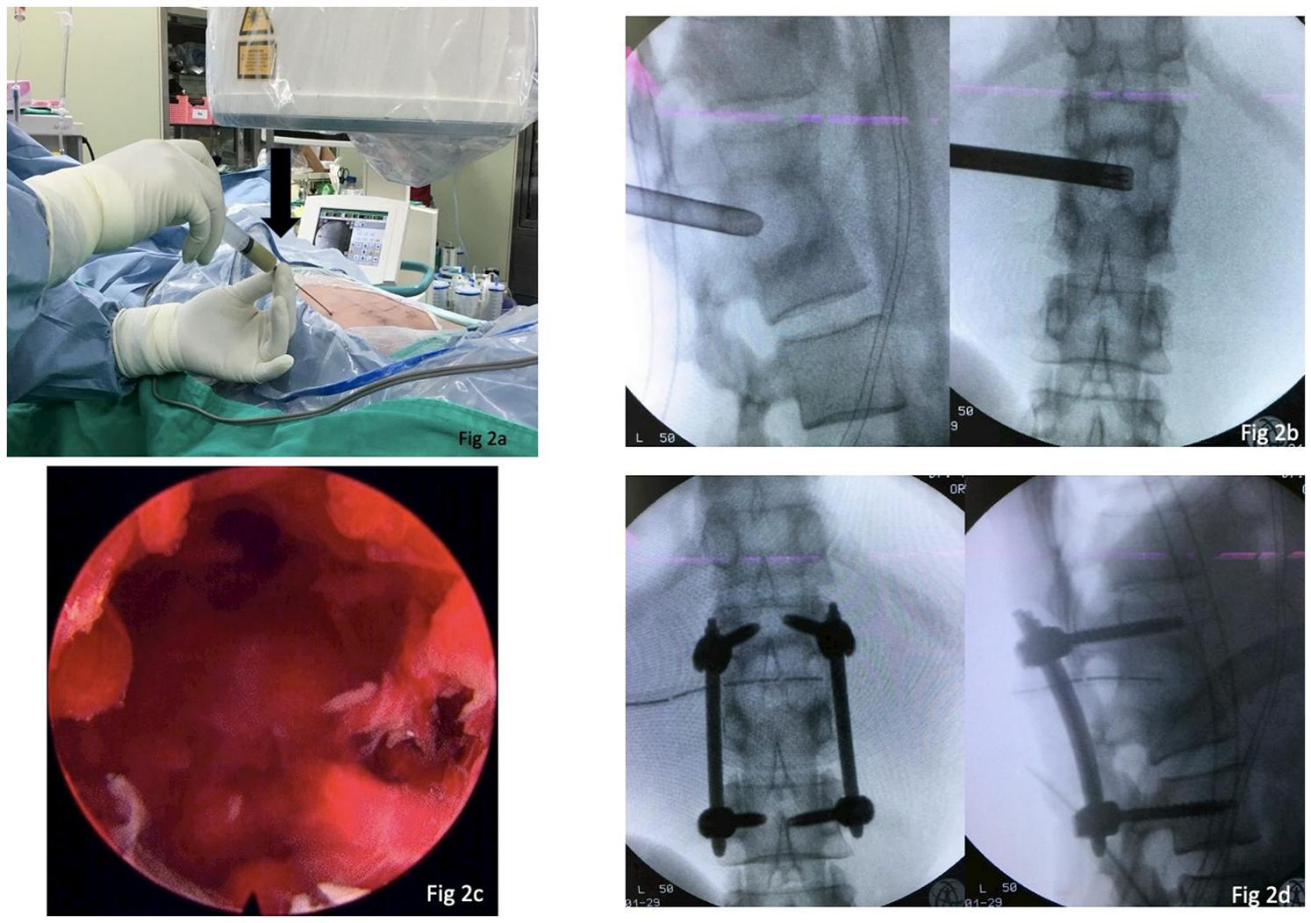
This patient underwent PEDD surgery. **(a)** First, a guide needle is inserted into the cavity via a left transforaminal approach, and aspiration of the abscess is conducted. **(b)** The working sleeve/trocar is inserted into the abscess cavity between the L1 and L2 vertebral bodies. Debridement and sequestrectomy are performed under endoscopy, followed by extensive normal saline irrigation. **(c)** The endoscope view reveals a cavity filled with fragile tissue and a hematoma after the abscess was drained. **(d)** Intra-operative fluoroscopy shows L1–L3 pedicle screws and a rod construct. Note that a 1/4-inch Hemovac drain is placed in the cavity.

**Figure 3:**
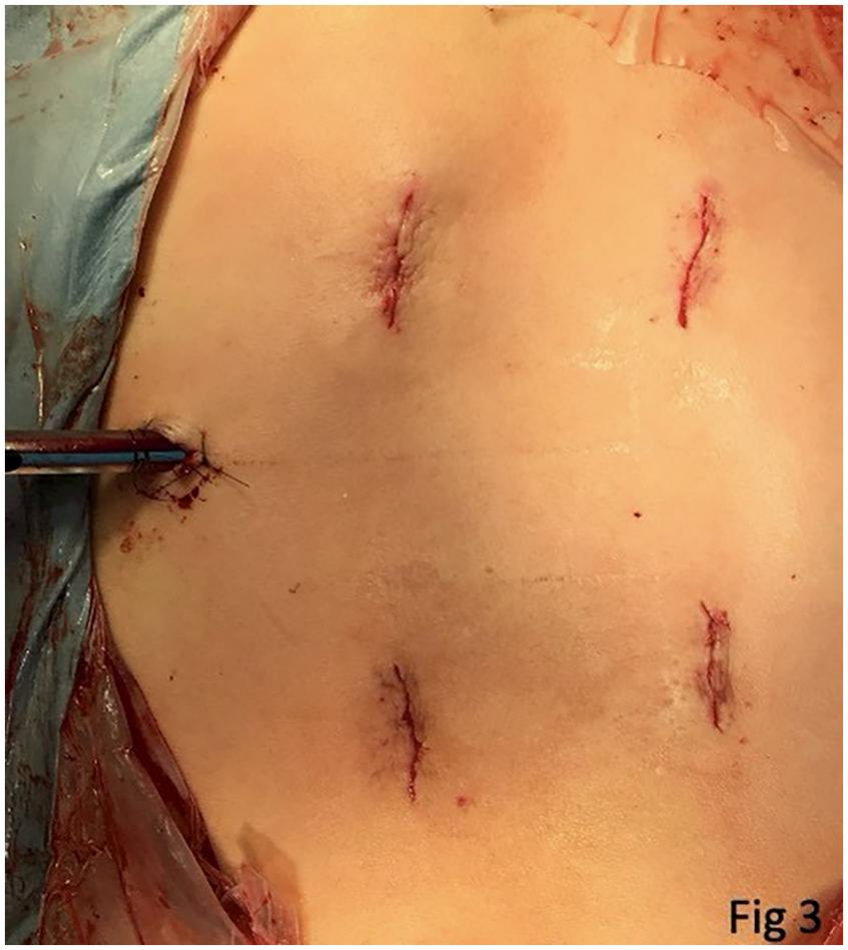
A clinical photograph of the patient’s surgical wounds reveals one wound (1 cm) for PEDD and four wounds (2.5 cm) for percutaneous screw insertion.

She underwent PEDD as a diagnostic and therapeutic procedure for the suspected abscess, with simultaneous minimally invasive pedicle-screw instrumentation. We adopted the transforaminal “inside-out” approach proposed by Dr. Anthony Yeung, which meant creating a working tunnel at disc level, approximately 30° horizontally in the axial plane. Most importantly, before the insertion of the working sleeve, long guide needle localization and aspiration should be conducted first (Figure 2a). Avoiding diluting the pus by irrigating it with normal saline may yield a higher culture rate. After aspiration to the highest available amount, the working sleeve was inserted via a guide wire into the abscess cavity. Debridement and sequestrectomy were performed using endoscopic instruments as cleanly as possible, followed by extensive normal saline irrigation (Figures 2b,c). After a 1/4-inch Hemovac drain was inserted into the cavity, we performed percutaneous pedicle screw insertion to prevent a possible fracture due to a weak posterior vertebral cortical bone (Figure 2d). There was one 1 cm wound for PEDD and drain tube insertion and four 2.5 cm wounds for screw insertion (Figure 3).

Fixation was primarily justified by the large two-level vertebral cavity and mechanical back pain indicating segmental instability, to avoid iatrogenic collapse and to enable early mobilization. Postoperative care was smooth, and her back pain subsided after surgery. The intra-operative culture yielded MSSA. Following infectious disease consultation, the patient was treated with intravenous oxacillin (2 g q4 h). The total duration of intravenous therapy was 4 weeks, and she was subsequently transitioned to oral antibiotics (amoxicillin/clavulanate) upon discharge. The patient could ambulate by herself 3 days after the operation and wore a back corset for 3 months.

Her CRP levels peaked at 12.006 mg/dL on postoperative day 4, declined to 6.590 mg/dL (day 7) and 4.298 mg/dL (day 14), reached 0.535 mg/dL at discharge (day 28), and normalized to 0.040 mg/dL by outpatient follow-up (postoperative day 57), paralleling steady pain relief and early ambulation.

**Figure 4:**
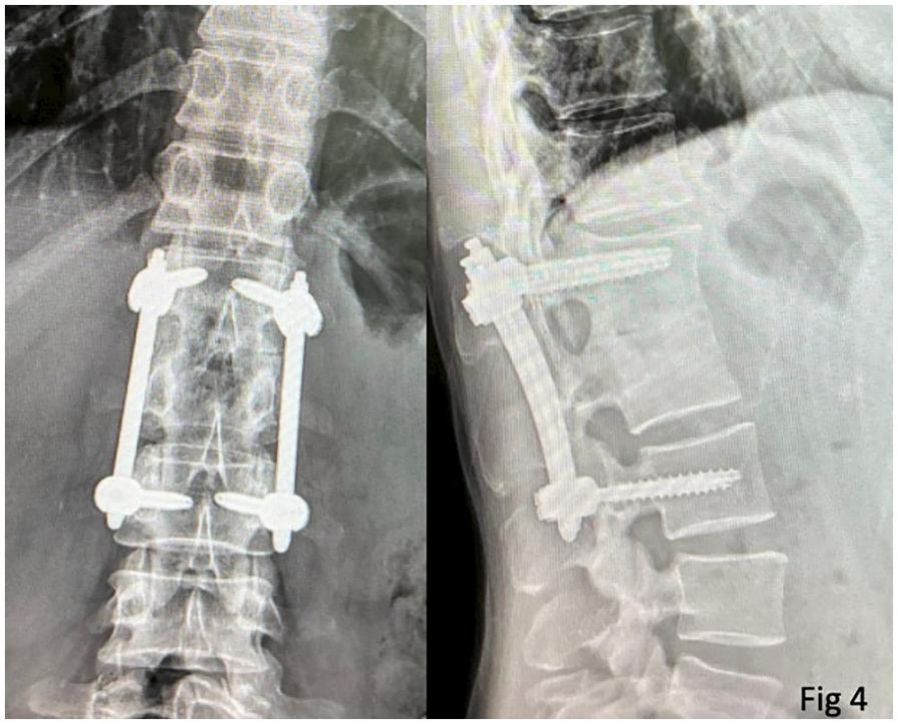
A radiograph of the patient’s thoracolumbar spine 2 years after the operation shows a fused L1/2 body and the absence of the previous cavity. The screws are well-fixed.

At the latest follow-up (2 years postoperatively), she could ambulate freely with slight back soreness. A radiograph of her spine revealed the union of the L1/L2 vertebral body, the absence of the previous cavity, and well-fixed implants (Figure 4).

## Discussion

A Brodie's abscess is characterized by subacute infection and chronic pyogenic osteomyelitis. In this case, the patient’s first visit for back pain was 2 years ago, in 2016, before admission. We hypothesize that the lesion began as a form of discitis. However, rather than progressing to typical acute pyogenic spondylodiscitis, the infection was likely contained and evolved into the subacute, localized Brodie's abscess within the vertebral bodies. This hypothesis would explain the chronic 2-year history and the distinct sclerotic rim seen on imaging (3, 5–7). The repetitive and chronic infection may have induced the fusion process between the involved vertebral bodies. Regarding the patient's significantly low body mass index (BMI = 13.6), this suggests a potential state of malnutrition. Malnutrition is known to adversely affect immune function. This may be an important comorbidity in this case, potentially increasing the patient's susceptibility to infections such as osteomyelitis, and could also pose challenges for postoperative recovery and tissue healing. The majority of Brodie's abscesses are found in the appendicular skeleton, usually in the tibia (48.6%) and femur (31.3%) (2). Brodie's abscesses have not been reported in vertebral bodies, except for a case reported by Lindsetmo in 1995 (7). In this case, imaging, including radiographs, CT, and MRI, was sufficient for diagnosis.

Surgical drainage is the preferred treatment choice for a Brodie's abscess when its sclerotic rim blocks antibiotic penetration. As for debridement surgery for an epidural abscess (8) and spondylodiskitis, it usually requires extensive soft tissue dissection, neural decompression (9), and even an anterior transpleural approach (10), which increases the risk of numerous complications (11).

In recent decades, endoscopic spinal surgery has been increasingly adopted to treat thoracic and thoracolumbar spine lesions, including herniated discs, canal stenosis, and even pyogenic vertebral osteomyelitis (12–16).

Several studies have shown that PEDD has the advantage of higher positive culture rates and better access to specimens for histological diagnosis (4, 16, 17). The wider endoscopic working channel (diameter 2.7–5.5 mm) allows for grasping forceps or other instruments to collect sufficient specimens (18). Yang et al. (19) stated that the endoscopic method yields higher positive culture rates compared to a CT-guided biopsy (90% vs. 47%). In our previous study, PEDD was proven to be a minimally invasive method with good safety and efficiency for treating a spinal infection (12). In this patient, whose abscess was located at the thoracolumbar junction, endoscopic surgery minimized the risk of approach-related complications compared to the traditional anterior thoracolumbar transpleural approach. She experienced minimal surgical wound pain and had an early recovery after the PEDD surgery. While a Brodie's abscess in the spine is exceedingly rare, traditional management principles for spinal infections, as cited in the literature, often involve invasive procedures such as anterior radical debridement and posterior instrumentation (20, 21). In contrast, our approach with PEDD achieved effective source control and stabilization through a minimally invasive pathway. This highlights PEDD as a viable alternative that may reduce approach-related morbidity and allow for faster patient recovery compared to traditional open surgeries.

Additional instrumentation with pedicle screws for spondylodiskitis may not be necessary in uncomplicated cases with a stable spine segment. However, in patients with segmental instability due to advanced vertebral body destruction, instrumentation has been suggested to provide better stability. In our case, given the significant two-level vertebral body destruction that compromised the anterior and middle column stability, concomitant instrumentation was indicated to provide crucial structural support, maintain spinal alignment, eliminate mechanical pain from the instability, and allow for safe, early mobilization.

Clinically, this case underscores that a rim-enhancing intravertebral cavity with a sclerotic margin often represents a contained nidus that requires source control beyond antibiotics alone; escalation from empiric therapy is warranted when pain or a high CRP level persists or when targeted cultures identify a treatable pathogen. Percutaneous endoscopic debridement and drainage can provide definitive source control with low morbidity and enable early mobilization, and objective stability criteria (e.g., Spinal Instability in Spondylodiscitis Score and flexion–extension radiographs) help determine the need and extent of selective instrumentation.

## Conclusion

In this case of a thoracolumbar Brodie's abscess, early recognition of the sclerotic-rimmed intravertebral cavity, the failure of medical therapy, and culture-guided endoscopic source control were key to the long-lasting resolution of the patient’s symptoms. Using objective stability criteria to individualize the limited percutaneous instrumentation enabled pain relief, early mobilization, and infection-free long-term outcomes. These principles may help clinicians decide when to escalate from antibiotics alone to minimally invasive debridement and drainage.

## Data Availability

The original contributions presented in the study are included in the article/Supplementary Material, further inquiries can be directed to the corresponding author.
